# Evidentiary vacuum, epistemic communities and rare disease policymaking in India: an evolutionary policy perspective

**DOI:** 10.1007/s10818-021-09322-y

**Published:** 2021-11-27

**Authors:** Shyamjeet Maniram Yadav, Saradindu Bhaduri

**Affiliations:** 1 Centre for Studies in Science Policy, School of Social Sciences, Jawaharlal Nehru University, New Delhi, India; 2 International Institute of Social Studies (of Erasmus University of Rotterdam), The Hague, The Netherlands

**Keywords:** Evolutionary economics, Evidence-based policy, Epistemic community, Rare diseases, India

## Abstract

There are divergent views among scholars and policymakers about the nature of permissible evidence for policymaking. It is often not feasible to construct a policy system exclusively based on objective research findings, particularly for rare diseases where conventionally accepted evidence remains a rarity. Evolutionary theories in such cases offer an overarching framework to represent the various heterodox understandings of what constitutes evidence and how evidence-based policies can be formulated under knowledge uncertainty. We conduct an empirical investigation of India’s rare disease policymaking endeavour in evolutionary perspective. The existing rare diseases policy architecture in India, in our view, reflects a ‘rationalistic’ framework. It intends to act only on ‘hard evidence’ to make, what may be called, an optimum decision, rather than initiating a ‘good enough’ policy decision based on existing (limited, soft) evidence and improving it incrementally through learning and trial-and-error. Our findings suggest that in the presence of ‘evidentiary vacuum’ and knowledge uncertainty, broadening the contours of epistemic communities, to include ‘lived experiences’ of the ‘lay’-stakeholders, can be effective in formulating an adaptive policy framework, which would ‘learn’ to better fit with the dynamic environment through inclusive deliberations, and trial-and-error.

## Introduction

The relationship between research and policymaking is a well-established topic of inquiry among policy studies scholars. More recently, the questions surrounding “hard scientific evidence” for policymaking have attracted intense deliberations in evidence-based policy (Young et al., 2002; Dobrow et al., 2004; Head, 2008). Amidst a growing interest in evidence-based policy, we find divergent views on what constitutes permissible evidence, the process of evidence generation, and its uptake in policy decision making (Sanderson, 2002). Nutley et al. (2013) argue that ‘scientific evidence’ describes information produced in a *particular* manner (method), broadly referring to information gathered *systematically,* through the use of *recognized* methods. In general, available literature on evidence-based policy looks at evidence as ‘something’—an outcome of ‘some kind’ of research. Different hierarchies of evidence have been developed to enable the ranking of research methods according to the validity of their findings (Evans, 2003).

In health policymaking, outcomes of the Randomized Controlled Trial (RCT), meta-reviews, systematic reviews, and expert opinions are often ranked hierarchically, in decreasing order of rigor and strength (O'Donnell et al., 2017; Naumova, 2017). This categorization, however, is not uncontested. The limitations of RCTs for not including what is already known (e.g. history), putting too much emphasis on unbiasedness over ‘what works’, and undermining clinical data are well known and pertinent in many situations (Jones & Podolsky, 2015; Deaton & Cartwright, 2018; Hortal, 2020).

In addition, a growing body of academic literature is highly critical of the idea that social policies can simply be ‘based’ on (hard) evidence alone (Marston, 2003; Head, 2010). Instead, evidence is only a component in policymaking, and such pieces of evidence are influenced by actors, contexts and the values of the decision-makers. Even decisions based on hard evidence are shaped by competing ideas and interests (Young et al., 2002). These arguments, in our view, provide compelling reasons to analyze the process of evidence-based policymaking from an evolutionary perspective.

An evolutionary framework is characterized by the unequal, uncertain, distributed, and developing characteristics of novelty creation processes, which include feedback mechanisms and the open-ended nature of development. (Mina et al., 2007). At its core, some of the building blocks of this approach are bounded rationality, diversity of agents, context and pathways, path dependence, trial and error based learning, group dynamics, and collective actions (van den Bergh & Kallis, 2013). Evolutionary thinking offers a dynamic view (at a dis-aggregated level) on how critical masses form and novel ideas and actions spread within a population (Witt, 2003). While many of the elements mentioned above have featured independently in several studies on policymaking, the evolutionary framework has the advantage of bringing them together under a single yet open, analytical framework, orthogonal to the discourse on policy processes based on perfect rationality.

Though evolutionary approaches have been used extensively in innovation studies, their use in policy scholarships remains few and far between. In addition, these studies largely remained conceptual or theoretical. Analyzing specific empirical cases through the lens of evolutionary mechanism have not yet been attempted adequately. The present study is an attempt in this direction, taking the recently initiated rare disease policymaking in India. We propose that the evolutionary policy framework can be used as an overarching framework to address some of the shortcomings of the discourse in evidence-based policymaking, especially when ‘objective evidence’ is in short supply and knowledge uncertainty is high.

A rare disease is perhaps a unique area where the conventionally accepted ‘evidence’ through large scale RCT, meta-reviews, and systematic reviews are hardly available (or even feasible to undertake). As a result, evidence-based policy formulation is an inherently challenging task in this area. Our study can, therefore, have implications for policy domains where a large amount of information is hard to come by, creating a dilemma for the policymakers on how/when to embark on evidence-based policy formulation. The findings of this paper can have policy implication for other developing countries, especially in South Asia. Many of these countries are yet to frame policy on rare diseases, though we witness a developing network of rare diseases stakeholders in Sri Lanka, Pakistan and Bangladesh.

Furthermore, epistemic communities have so far been studied from the perspective of analyzing their role in policy learning and policy transfer. The process of their formation and the way they shape evidence in a field where hard evidence is not easy to come by, have not found a place in the relevant scholarship so far in developing countries. We also observe that the institutionalization of patient support and advocacy groups in policy decision-making remains inadequate in these countries. By epistemic community, these countries predominantly mean the views of the formal experts. The importance of patient support and advocacy groups in this framework does not hold much importance. We argue how these groups contribute to enrich context-specific knowledge in policy processes. This way, their inclusion within the broad category of the epistemic community might directly bear on the efficiency of policy decision-making.

The paper is written at the backdrop of the current policymaking process for rare diseases in India. While the need for a policy is being increasingly recognized by diverse groups, its pathways, and the required policy steps have become a subject of controversies and disagreements. While the government wants more ‘evidence’ to initiate the policy process, patient support and advocacy groups, supported by local physicians, claim that sufficient ‘evidence’ does exist to initiate the process. Similar disagreements prevail over the likely policy steps. While researchers want more policy support for basic research, physicians, pharmaceutical companies, and patients support groups make a pitch for immediate patient care support and import of drugs. In the Indian health policy setting, these groups have very different standing, and preference for what constitutes ‘evidence’. All this has made it difficult for the policy process to unfold. As a consequence, a policy draft was introduced in 2017, taken back in 2018, and reintroduced, at the insistence of the Court, again in 2020 without further progress.

Next, we present a framework of evolutionary policy scholarship. Here, the literature on epistemic communities and their role in evidence formation has also been explained. The subsequent section gives a brief account of the rare diseases policy instruments across the globe. We then move on to explaining the data and methodology for the current research. Next, we discuss in detail the observations from the field and presents our analysis. We then make our concluding remarks.

## Evolutionary policy perspective: the broad contours

The evolutionary approaches are orthogonal to the so-called rational choice models of policymaking. Rational choice theorists advocate that decision-makers understand the meaning of the choices they confront; their only problem is to select, from among the possible known actions and options, the optimal ones given their preferences and the constraints imposed by scarcity (Gifford, 2005). However, taking cues from the concept of bounded rationality, evolutionary scholars emphasise on subjective interpretation, and incomplete understanding, of the environment, while making decisions (Simon, 1990; Landa, 2002). Imitation, habitual behaviour, and myopia could therefore be prevalent when agents are boundedly rational (van den Bergh & Kallis, 2013).

Witt (2003) argues that political actors or policymakers are not omniscient. They do not possess all the information at a particular time. They learn along the way, behave according to adapted (or selected) habits and routines, imitate others, and are myopic (in time and space). The myopia might also shape selective filtration and retention of information, and make their learning non-linear (Epp, 2017). The policymaking process, as a result, evolves through trial and error, learning, and adaptations (Witt, 2003). Consequently, bounded rationality leads to diversity in knowledge, beliefs, perceptions, and approaches among the policy actors. The policy making, under such environments, “proceeds in a series of fits and starts, with long period of statis, interrupted by brief but dramatic periods of change” (Epp, 2017, p. 54).

Due to this centrality of bounded rationality in the evolutionary framework, we first present the nuances of bounded rationality before proceeding further.

## Bounded rationality and uncertainty: a brief overview

In the proposal of Simon (1990) the framework of bounded rationality is shaped by a pair of scissors, whose two blades are ‘the structure of task environments and the computational capacities of the actor’. In particular, actors suffer from limited cognitive ability in a dynamic decision environment, making it almost impossible to have full knowledge about the environment, and the decision tasks, as envisaged in the theories of rational choice. Temporally too, due to the changing nature of the environment, an important component of the task environment is uncertainty in terms of how well a criterion can be predicted (Todd & Gigerenzer, 2007).

The distinction between risks and uncertainty are important to note in this regard. In the framework offered by Frank Knight an unknown feature can be formally characterized and assessed in the form of three probabilities- a-priory probability, statistical probability and estimates. A-priory probability corresponds to propensities, and not on observations (type-1 risk) Statistical probability is calculated based on empirical data from repeated experiments under homogeneous environmental conditions. Third type of predictions is estimate, which work best in case of uncertainty related to the real-life problems. Statistical probability would be insufficient to capture such outcomes, given the dynamic nature of the environment. The latter type of problem situation is defined as uncertainty, while the former two types conform to the situations of risks. For Knight, most of the decisions in the real world, are based on estimates (Mousavi & Gigerenzer, 2014).

Limited cognitive capabilities and uncertain environment pose a challenge for the policymaker to adopt a perfect rationality worldview to policymaking, since hard objective evidence are not only scarce, but could also be of limited relevance in a long term policy process under changing task environment. Heuristics, judgment and gut feeling of the decision maker play a crucial role under such circumstances. Subsequent research on bounded rationality have taken two, purportedly non-overlapping pathways.

The heuristics and biases school, propounded by Amos Tversky and Daniel Kahneman acknowledge the presence of such tools of decision-making, but point out that the decisions based on these tools are often ‘biased’, and largely remain inferior to the ‘optimum’ decisions arrived at by the rational calculations.^1^ Their arguments have, however, been sharply contested by several scholars. Putting forward the so called ‘fast and frugal’ narrative, Gerd Gigerenzer and his colleagues argue that biases may not be undesirable under situations of uncertainty, and intuitive reasoning might work better than rational calculations, in the presence of ecological rationality of the given decision context (Gigerenzer & Gaissmaier, 2011). These scholars further affirm that in a dynamic and uncertain environment, the decision maker does not opt for optimization; rather they look for the “satisficing” solutions and their incremental adaptations. Through the entire process, the decision-makers would apply heuristics to make judgments in complex decision-making, using simple, indirect cues (Bergan & Fitzpatrick, 2021). The heuristics may, for instance, come in the form of what Gigerenzer and Gaissmaier (2011) refer to as ‘one-reason decision making’, where generalized cues are drawn based on socioeconomic status, geography, and perceived needs of the communities. With sufficient experience, people learn to select useful heuristics from their adaptive toolbox.

## Evidence in policy analysis: objectivity, hard evidence, and evolutionary processes

Conventionally, in evidence-based policymaking, Randomized Control Trials (RCTs) have been widely recognized as the gold standard for evidence generation. The support for the RCT among the policy scholars comes from the fact that it is experimental, reduces bias, and controls personal views and prejudices from diluting the policy environment. In many places, it has also helped effectively counter policy paralysis arising out of non-agreements of experts (Goldacre, 2013). The assumptions that randomization and blinding may remove such biases has led to its popularity and wide application in diverse fields of applications.

Despite these merits, RCTs do have limitation in cases where knowledge uncertainty is high. Its limitation to include what is already known (e.g. history), putting too much emphasis on unbiasedness over ‘what works’, and undermining clinical data are well known and pertinent in many situations (Jones & Podolsky, 2015; Deaton & Cartwright, 2018; Hortal, 2020).

Deaton and Cartwright (2018) have pointed out that RCTs, over time, have drifted from their original focus on ‘internal validation’, to put more emphasis on the ‘external validation’ of the results, even when the ‘portability’ of such results was weak. This (over-)emphasis on external validation may undermine the need to account for rich context-specific information and social norms in designing its framework. Moreover, an RCT interprets the results in terms of probability, and *not* frequency. It has been observed that people do not ‘experience’ probability, and, therefore, may misinterpret the results of RCTs when they are presented in terms of probability. In contrast, people ‘experience’ frequencies in their daily lives, and can more effectively interpret results expressed in terms of frequencies (Wheeler, 2020).^2^ In so far as ‘context specificity’, and ‘experience’ are the cornerstones of ‘ecological rationality’ in a bounded rationality paradigm, the framework of RCT seems to be at odds with such a policy environment.

The evolutionary framework to policymaking recognizes diversity in the sources and nature of evidence, going beyond the conventionally accepted understanding of evidence—exclusively as an outcome of systematic research (Stevens, 2007). Here, evidence may appear in the form of facts, findings, or recommendations produced not only by researchers but also by ‘journalists, think tanks, pressure groups or others’ (ibid). Recent research suggests that the inclusion of more qualitative research from the grey literature should be brought to the domain of evidence (Harder et al., 2015). Stevens (2007) argues that the evolutionary models allow examination of how (and which) evidence shapes the climate of opinion in which a policy is made. Furthermore, research evidence itself is often either absent or contradictory. As a result, value judgments that cannot be decided by research alone will always play an important role.

It is well established that knowledge is distributed within and across communities, linked through a variety of formal and informal mechanisms (Mina et al., 2007). The diversity of information and knowledge required, and values involved, in the policymaking process makes it necessary for policymakers to rely on what is known as ‘epistemic community’. Epistemic communities are a “network of professionals with recognized expertise and competence in a particular domain and an authoritative claim to policy-relevant knowledge within that domain or issue-area” (Haas, 1992). These communities broaden the ambit and definition of ‘evidence’ and induce learning among policymakers. In the presence of the diversity of actors in a particular policy space, epistemic communities steer the policy directions, enable policy action and consensus-building (Haas, 1992).

Witt (2003) also visualizes the emergence of social networks and communities with similar-minded political actors. Hibbert et al. (2016) extend this line of research further, by analyzing the way collaborative understanding is shaped through shared practices among established, yet epistemically disparate communities. Such formations are shaped by recursive feedback between perceptions, attention, and preferences of boundedly rational agents having highly intensive internal communication, while remaining relatively insulated from external influences (Witt, 2003). Regardless of the professional background, epistemic communities have a shared set of normative and principled beliefs, which provide a value-based rationale for the social action of community members (Hass, 1992).

Interestingly, these communities do not only promote diversity in actions and thoughts; they bring experts together explicitly to develop new knowledge and display a high propensity to innovate (Amin & Roberts, 2008). The epistemic community’s participation in the policymaking process broadens the domain of what is constituted as evidence by bringing in their ‘perspectives’ and ‘insights’. The claimed monopoly over a specific body of knowledge the communities bring in can, therefore, have a bearing on the policy trajectory. The interactions between them and policymakers, however, do not happen in a vacuum. It occurs in the continuously changing economic and political environment, which keeps throwing new information and knowledge.

In so far as the members of these communities are often responsible for generating new information and scientific knowledge, they continue to enjoy an edge over policymakers in shaping the policy trajectory. There is a strong incentive for the groups with diverse interests, knowledge, and values to participate in the communication and agenda-setting phase of policymaking, leading to a complex process of multistage propagation and negotiations involving legislative representatives and bureaucrats (Witt, 2003). Furthermore, it is common to find that collaborative activities at one level lead, directly or indirectly, to activities at other levels, which gives these processes an evolutionary character (Imperial, 2005).

## Rare disease policy instruments: a brief review of policies across countries

The estimated number of globally known rare-diseases vary between 6000 and 8000, most of which fall into the domain of pediatric disorders (Dodge et al., 2011; De Vrueh et al., 2013). Though the cumulative number of the whole spectrum of rare diseases is large (more than 350 million), the number of people affected by any specific disease often remains very low. The rarity of these particular diseases manifest into problems in terms of (a) designing clinical studies^3^; (b) funding of research programs; (c) the discovery, testing, and approval of new treatments; and (d) the training of clinical scientists (Griggs et al., 2009). Further, rare diseases presumably affect only a fraction of the population in an area, are non-contagious, do not cause immediate mortality, and shows delayed severity. Therefore, the conventional ‘public harm’ based rationale for policy intervention cannot be invoked easily in this field.^4^

Indeed, globally, any systematic policy intervention to deal with rare diseases began only in 1983, and comprehensive policy efforts, even today, largely remain confined to the countries in the global north. Since the enactment of the Orphan Drug Act in 1983 in the United States of America (USA), countries in Europe and, of late, a few Southeast Asian countries have adopted various policy efforts in this domain. The policy instruments for the rare diseases are broadly concerned with—(a) developing diagnostics/genetic testing/genetic screening; (b) research and development to develop new drugs; (c) regulatory approval and market access to existing drugs; (d) access to treatment/reimbursement of existing drugs and treatment regimens under Orphan Drug Policy, and (e) creating registries (Table 1). We observe certain inter-country variation too in the selection of policy instruments. Some countries have a well-developed, comprehensive, and coordinated approach to diagnose, treat, and manage such diseases. In contrast, others have used only a sub-group of the instruments mentioned above, primarily aiming at the management of the diseases (Table 1).

**Table 1:** Overview of rare disease policies across countries

Country	Policy stage	Registries	Neonatal screening	Genetic testing	Orphan drug legislation	Orphan drug reimbursement	Orphan drug incentives	Orphan drug in the market
Canada	Adopted/implementing	Development stage	Yes	Yes	Accelerated review	In some cases	In the process	Yes
USA	Fully implemented	Yes	Yes	Yes	Yes	Yes	Yes	Yes
Russia	Implementation stage	Yes at the development stage	Yes	Yes	Yes	Yes	Yes	Yes
United Kingdom	Formulated	Yes	Yes	Yes	Only at European level	Yes	Yes	Yes
France	Formulated	Yes	Yes	Yes	Yes	Yes	Yes	Yes
Germany	Formulated	Incipient stage	Yes	Yes	Yes	Yes	Yes	Yes
Italy	Formulated	Yes	Yes	Yes	No	Yes	Yes	Yes
Netherland	At implementation stage	Yes	Yes	Yes	Yes	Yes	Yes	Yes
Denmark	Discussion stage	No public registries	Yes	Yes	No	No	Yes	Yes
China	Developing guidelines	At the development stage	No	Yes	No	No	No	Yes
Japan	Programmes	Yes	Yes	Yes	Yes	Yes	Yes	Yes
Taiwan	Implementation stage	Yes	Yes	Yes	Yes	Yes	Yes	Yes
South Korea	Yes	Yes	Yes	Yes	Yes	Yes	Yes	Yes
Switzerland	Yes	Yes	Yes	Yes	Yess	Yes, but no market exclusivity	Yes	Yes
Singapore	Formulation stage	Yes	Yes	Yes	No publically available information	Yes	Yes	Yes
Norway	No	Yes	Yes	Yes	Yes	Yes	Yes	Yes
Estonia	Yes	Yes	Yes	Yes	No	Yes	Yes	Yes
Australia	Recently action plan	At the stage of development	Yes	Initiated	Yes	Yes	Yes	Yes
Malaysia	In the process	No	Yes	Initiated	No	No	No	No

*Source* Our compilation

For many countries in Europe, we rely on Rare Disease plans and strategies in European countries, which can be accessed on https://www.eurordis.org/content/rare-disease-plan-and-strategies-european-countries. Last accessed on 22 June 2020

Among all the countries we have surveyed, the countries in EU have a well-articulated, multi-pronged strategy in this regard. They have developed a pan European network, namely, the European Reference Network (ERN), to diagnose, research, and treat various rare diseases. These countries have developed registries at multiple (continental, national, regional, and local) levels for data collection. One crucial feature of the policies in the USA and Europe is the extensive engagement of the established patient advocacy groups in rare disease management. These groups have national as well as regional presence representing specific diseases. The USA and the European countries have developed an extensive formal institutional mechanism to involve such groups and patients in the active policymaking arena.^5^ These patient advocacy groups bring experiential knowledge that complements the scientific evidence for rare disease policymaking. More recently, South East Asian countries too have initiated policies in this area. However, the instruments of these countries are largely confined to accessing orphan drugs. Registries and genetic screenings remain unevenly distributed across these countries. Even concerning the access to therapy, we do not find systematic attempts in these countries so far.

## Data, sample, and method of study

### Data sources

We conducted interviews with twelve patient advocacy groups, five clinical practitioners and two clinical geneticists from King Edward Memorial Hospital (KEM, Mumbai), JK Loan Hospital (Jaipur), Sir Gangaram Hospital and All India Institute of Medical Sciences (AIIMS, New Delhi), and four basic science researchers involved in the making of the policy. We also took interviews with three representatives of the multinational pharmaceutical firms engaged in rare disease research. Specific semi-structured questionnaires were used to conduct these interviews.

Besides, we participated in various round-table discussions and conferences on rare diseases policymaking. These meetings took place at Jawaharlal Nehru University (JNU, New Delhi), Indian National Science Academy (INSA, New Delhi), and the India International Centre (IIC) in New Delhi. It is worth noting that these meetings were attended by a diverse group of people, including social scientists, parents of affected children, caregivers, government representatives (e.g., officials from NITI Aayog^6^), lawyers, patient advocacy groups, and clinical practitioners.

The study has also drawn upon policy documents, court rulings, and Parliament deliberations.^7^ At times, we had to rely on responses to queries filed through the Right to Information Act (RTI) for certain information from the government departments. All this information was collected during February 2017–June 2019.

### Data collection: strategy and process

For primary data, respondents were first contacted in a gathering of stakeholders in 2017 at JNU. Further identification of actors was done through snowball sampling. The sampling also revealed the complex, evolving network of patient advocacy groups, clinicians, and researchers. These networks have helped us discern the pattern of interaction among the stakeholders. The mutual differences, conflict, and disagreements among the stakeholders also provided us with essential insights into stakeholder identifications, group formation, propagation for critical mass, and the interest dynamics.

After establishing the initial contacts, each respondent was explained about the researchers’ identity and the objective of the interview. Very few respondents gave their concurrence to audiotape the conversations. Commonly, the interviews began with a few questions on their previous activities. It was during this discussion that most of the policy-related issues emerged. Subsequent questions were about the specific diseases they are dealing with, their interaction with other stakeholders, the sources of finding, and their views on, and expectations from, the policies. Some of the interviews were difficult, mainly when the respondents were also the parents of the patients. The interviewer tried not to engage with the personal aspects of the disease management they were involved with if the respondents were not bringing it on board themselves. The questions were mostly focused on the emergence of the patient advocacy groups, their interaction, difficulties they encounter, and their policy-related knowledge. Most patient advocacy groups (barring two) were happy to share information.

The collection of data from the policymakers and people directly involved in the policymaking process has been a challenging task. Responses from the government organizations were hard to come by, prompting the researchers to use the Right to Information (RTI) as a data collection tool. We used the RTI mostly to obtain information regarding dates and frequency of, and participation in, the various meetings held in the government departments. Judicial orders and deliberations, arguments presented in the various Public Interest Litigations (PIL) have been an important source of data too.

Several secondary sources of data have also been used. These sources include company websites and reports, patient advocacy groups, government institutes such as the Ministry of Health and Family Welfare (MoHFW), Indian Council of Medical Research (ICMR), news publications and interviews published on various platforms. Websites of the patient advocacy groups helped us trace the contribution of each patient advocacy group. The patient advocacy groups have mentioned in detail the chronology of events they have been part of, along with the media coverage of these events.

Since the study is a policy analysis, and we did not intend to extract personal or disease-specific personal information, an approval from the Committee of Advanced Studies and Research (CASR) of the School of Social Sciences of the University was necessary and sufficient to conduct the research. As explained above, names or personal information have been withheld in the analysis.

Data thus collected has been analyzed thematically. We first created the key thematic categories, and then analyzed the themes by processing the data manually. A major limitation of our data is the lack of detailed explanation from the policymakers about the various internal mechanisms of the policy process. A few major patient advocacy groups too were reluctant to divulge the detail about their interaction with the industry houses engaged in drug development. We, however, attempted to analyze these non-responses too.

## Observations and analyses

Despite reports of more than seventy million population of India being affected by rare diseases, the public attention received in the media, academic scholarship, and policymaking remain inadequate. While the recent policy draft has raised optimism among several stakeholders, reports published in the newspapers and magazines have brought to the fore certain important concerns regarding the policy process, its goals, and its feasibility, leading to its delay and inertia in its implementation. In short, the making of the rare disease policy witness multiple back-and-forth movements, inertia in resource allocation, and path dilemmas. In this section, we analyze the government’s claims about (non-) existence of evidence needed for a comprehensive policy for rare diseases. In particular, how the diverse set of actors bring their epistemic ideas, values, and interest about what constitutes evidence is the issue we deal with before proceeding further.

### The evidentiary vacuum: claims and contestations

The government is insistent on the non-availability of epidemiological data as the key reason for the delay in policymaking. Subsequently, it was agreed to set up a rare disease registry in 2017 to collect epidemiological data (e.g., ‘evidence’) needed to frame subsequent policy instruments. While the major stakeholders have widely accepted the choice of this instrument as a first step, the registry remains to be operational, till date. The lack of resources to form the registry and its related regulatory infrastructure is, allegedly, causing this policy inertia.

At various meetings or roundtable discussions, officials of the government departments insisted on more extensive epidemiological information. This is not surprising, as evidence in health policymaking conventionally emphasized RCT outcomes, meta-reviews, and systematic reviews to frame policy instruments. This phenomenon reflects a typical path dependency in resource allocation, where allocation to ‘new resource heads’ faces more significant difficulties. The demand for more evidence gets support also from the prominent basic science researchers involved in the deliberation. They keep demanding enhanced grants and regulatory flexibility to undertake experiments on vector construction, gene therapy, and small molecules trials in the areas of rare diseases to bridge this knowledge gap.

On the other hand, many stakeholders believe that enough ‘evidence’ is available for initiating, at least, the process of treatment and reimbursement of medical costs. These stakeholders include patient support and advocacy groups, clinical practitioners, and business firms.^8^ Further ‘evidence’, according to these actors, could be generated in a ‘step-by-step’ manner through the registry itself. A prominent business firm involved in rare disease drug development is of the view that the government is reluctant to take concrete steps to set up the diagnostic facilities to keep the prevalence of rare diseases ‘below the radar’, thereby avoiding future resource commitment in this area. They, however, think that credible information in this regard is scattered and can only be accumulated if the policy broadens the ‘catchment area’ of data collection beyond the conventional groups and sites.

Conventionally, the collection of policy-relevant data have not included patient advocacy groups, and sites other than the tertiary government hospitals. In rare diseases, on the other hand, an extensive repository of information is available with the various patient’s support and advocacy groups, and in large and small private hospitals. Independent clinicians too possess important information on such diseases. A departure from the existing policymaking routines is thus warranted to initiate the process of rare disease policymaking, suitably. In other words, the policy dilemma and inertia that rare disease is currently facing has its reasons rooted in the historical contingencies that seems to have shaped the path of health policymaking in India.

In addition, the patient advocacy groups claim that they, along with the clinical practitioners, have enough evidence regarding rare disease caregiving, which should be brought into rare disease policy too. Some of these groups have developed a network of multidisciplinary teams to manage specific diseases on their own, in the absence of a policy. For instance, the Organization for Rare Disease India (ORDI) and CureSMAIndia, have made a protocol for diagnosing and managing the rare diseases they are concerned with. Patient advocacy groups have developed mechanisms for gathering information, generating awareness, and collaborating with the other stakeholders engaged in diagnosis, treatment and management. We can observe an ecosystem of rare disease management being developed where each stakeholder is equipped with the experiential knowledge from her own ‘lived experiences’. However, the government remains insistent on the conventional form of hard evidence of epidemiological data for resource allocation and reimbursement of treatment costs, giving rise to policy inertia and discontinuity.

### Evidence making: negotiation and contestation

As pointed out the diverse actors involved in this process differ quite considerably, in terms of their preference for policy instruments. The judicial intervention, in this context, has an important bearing on the rare disease policy outcome and learning. In fact, due to the imposed time limit by the Judiciary, the first draft was framed without much deliberation. Field interviews revealed that there was limited information available in the public domain, about policy formulation, in this phase.

An RTI to the Ministry of Health and Family Welfare does not reveal much on the deliberation for preparing the policy draft in 2017. Stakeholders were engaged only with the second draft, which was unveiled in January 2020. Prominent stakeholders-the Lysosomal Storage Disorder Society (Delhi), ORDI (Bangalore), MERD (Jaipur), Indian Organization for Rare Disease (I-ORD) appeared to be most active in policy deliberation at this stage. These groups have a strong network with the Researcher at JNU, IGIB, clinical practitioners at AIIMS (Delhi), KEM (Mumbai), and JK Loan Hospital (Jaipur).

The industry predominantly wants fiscal incentives for manufacturing and purchase of drugs. We found that firms are willing to collaborate with the basic science researcher too, to research on vector constructs, which could pave the way for gene therapy. In this regard, there was also the discussion on making a consortium of companies, basic science researchers, and clinical practitioners to introduce gene therapy in India for a select group of diseases. With the help of basic science researchers and other stakeholders, the industry expects to obtain certain regulatory relaxation for expeditious drug development in these fields. Researchers too demand more significant funding and regulatory provisions for gene therapy trials or small molecules trials in the specific disease areas they are working in. Some such demands have been fulfilled, particularly when similar examples are available from elsewhere.

Clinical practitioners are primarily concerned with the data from their diagnoses, based on the symptoms or disease specifications. However, the cooperation between basic science researchers and clinical practitioners remains inadequate. A clinical geneticist revealed that they do not want to share the data with others unless there are incentives for publications and recognition. Similarly, many small and scattered groups of clinicians too prefer to hold on to the private registries they have created, as a step to ensure ownership of data. Interviews with the clinical practitioners revealed that the absence of a clear mandate from the government has given rise to the growing tendencies among the smaller groups to develop their own data and networks. At times, the absence of an acceptable format for data storing/sharing has been held responsible for inadequate data sharing in this field too. Besides such demands, clinicians favour the idea of improving the diagnostic facilities and create caregiving infrastructure. They also emphasize the need to include rare diseases in the medical curricula. Their demand, unlike that of the scientists, however, remain mostly unaddressed in the policy deliberations. In our view, one cannot undermine the rich experiential domain knowledge of these actors (the patient support groups, companies, or clinical practitioners), their varied interests notwithstanding. These experiences, unfortunately, remain under-exploited largely due to the over-emphasis, of the policymaking bodies, on ‘hard evidence’.

### Policy trajectory: deliberation and uptake

The deliberation and debate over the policy goals have taken place at various formal and informal platforms. These deliberations and the discussions involve, mainly, epistemic communities from prominent institutions. While much of these deliberations have revolved around the collection of epidemiological data and possible treatment processes, the patient advocacy groups, have pointed out the need policies to generate awareness through creating social platforms of collective actions and information sharing.^9^

The insistence on hard evidence is perhaps entangled in a nuanced articulation of group interest, lived experiences, and policy learning from other countries. A leading patient advocacy group member, in this context, reveal that while the lack of hard evidence is being seen as the main reason for policy initiation, it did not deter the major interest groups to agree on the broad number of patients in the country. In his view, the number of rare disease patients in the country was arrived at using estimations and extrapolations, and not by any concrete calculation. The frequently quoted figures of 70 million patients and 7000 diseases are only approximations, not based on any hard scientific evidence either.

The definitional issues around what constitutes rare diseases indeed remain unsettled. While the researchers seem to favor a relative measure (such as the benchmark of 1 case per 4000 population) to qualify a disease to become a rare disease, the larger patient advocacy groups suggest the benchmark should be in terms of absolute number and suggested 5000 to be the cut off in the country. The groups representing “ultra rare” diseases (i.e. smaller number of patients), however, expect the benchmark to become 2500. The government appears to be open to any benchmark, but only after collecting extensive data, the format of which is currently being operationalized by the Indian Council of Medical Research (ICMR).

Proposed policy instruments in the draft policy are concerned with creating large governance structures with long-term consequences. While it is important to build the institutional architecture and facilitate research and development, we would have to admit that the outcomes of such actions are ‘uncertain’ and only realized with a long gestation period. Furthermore, creating such architecture would require a consistent allocation of resources for a considerably long period. Patient support and advocacy groups, on the other hand, remain in a hurry to find some policy relief to their immediate needs of caregiving and patient management. They feel their demands for treatments, caregiving, reimbursement and management remain marginalized in the policy deliberations. They also point out that the exclusions of data generated out of ‘lived experiences’ are at odd with the global pattern of policymaking observed in this area.

As indicated above, the collective actions are sporadic and confined to several smaller groups that do not communicate much with either the larger groups or the government. In our interviews, many such small groups and networks reported their reluctance to share data (biological samples, epidemiological data) with the either the public or the government due to the former’s non-inclusion in the policy process. Instead, these networks are eager to share their data with the firms involved in drug development.

Along with scattered knowledge bases and the emergence of multiple networks, we observe that stakeholder deliberations are fragmented. There is no forum or interactive platform where government and stakeholders can come together for systematic deliberations. Such reluctance to involve the patient advocacy and support groups raises an important theoretical question on what should constitute an epistemic community. Conventionally, epistemic communities comprise professionals such as scientists, academics, and physicians who rely on the various aspects of objective scientific evidence. Lived experiences, it appears, do not qualify as knowledge of good enough rigor.

The Indian policy dynamics raises a question on the viability of this hierarchy of evidence. Discourses on scientific knowledge too admit that scientific knowledge can be subjective, site-specific and cultural (Livingstone, 2010). Ignoring site-specific experiential learning would adversely affect policymaking, especially when “hard objective evidence” does not seem to be available. Indeed, we observe that the first rare disease policy draft mainly compiled the recommendations by the department officials and committee of experts largely drawn from medical practitioners. Subsequently, however, the draft had to be rolled back. Another draft has been framed but based only on limited (and perhaps inadequate) consultations, and only after the judicial intervention, but only to be shelved, once again. Clearly, the drafts have failed to generate the critical minimum level of agreements among the stakeholders needed for their implementation.

## Discussion and concluding remarks

It is often difficult to obtain the so-called “hard” objective evidence, considered to be the gold standard for evidence-based policymaking, in the field of rare diseases, simply due to the rarity of the occurrences of such diseases. The fragmented nature of the patient population across diverse cultural and geographical settings too weakens the scope of ‘external validation’ of the results of RCT, and, hence, their applicability across settings. Moreover, high possibilities of differential diagnosis, absence of standard protocols of treatment, and uncharted disease pathophysiology raise uncertainty in the governance of rare disease, compounding the difficulties of deploying the methods of rational choice for policymaking in this field.

We made an effort to engage with evidence-based policymaking through the lens of evolutionary ideas and thoughts, which embrace a broader meaning of what constitutes evidence than the orthodox rational choice theories. This framework embraces expert’s opinions, individual judgment, and ‘lived experiences’ as evidence along with the so-called hard evidence of scientific research. We argued that evolutionary ideas could be used to offer an overarching framework for the various heterodox understandings of what constitutes evidence and how evidence-based policies can be made under knowledge uncertainty. How do epistemic communities emerge and collaborate in this context then becomes an important and hitherto unexamined point of inquiry too.

Conventionally, health policies in India have been made with the help of expert’s knowledge and available ‘hard evidence’. Decisions on allocating health resources at both the national and state levels are predominantly based on consensus opinion from expert committees (Downey et al., 2017). Moreover, in Indian context, the expert’s knowledge has predominantly been drawn from medical and public health professions, that too from those belonging to reputed institutes and large tertiary care hospitals, with minimal roles of primary care physicians or patients support and advocacy groups. Ironically, in rare diseases, it is the second group, which possesses the most amounts of data. While the governments do not rely exclusively on hard evidence and are open to using subjective experts’ knowledge for policymaking, they remain reluctant to accept subjective evidence from the so-called ‘layperson’s perspectives’ of the patients’ groups and even primary care physicians.

The policy architecture, it appears, is driven by the assumptions of omniscience of policymakers, and yet to acknowledge the presence of bounded rationality among policymakers and experts. Consequently, the policy drafts are leaning towards treatment, epidemiological data collection and laying out the research pathways, ignoring the immediate goals of creating awareness, caregiving and management. There is a denial to embark on an incremental policy process through trial and error and learning. Instead, the government prefers to develop an extensive database of ‘hard evidence’, before embarking on policymaking. To us, this dilemma reflects a ‘rationalist’ frame of mind, where ‘good enough’, ‘satisfying’ decisions are ignored in want of the ‘optimum’ (Patil & Bhaduri, 2020). However, such delay can prove to be counterproductive by discouraging the excluded groups from pooling the data and evidence they have for public use, besides of course, delaying the framing of the policy itself.

There are indeed instances where patient advocacy groups have contributed to the management of the diseases through their understanding of the context. We found that a few actors successfully improvised solutions to unmet problems, based on experiential knowledge and ecological rationality. Administration of soya milk for Lactose intolerant patients of Pompe diseases could be referred to as one such example. Subsequently, soya milk has become a well diffused protocol to manage the disease across the board.

We find that many epistemic communities have been formed to influence the policymaking exercise in recent years, driven both by professional interests of researching uncharted areas and social commitments. These communities are helping to build the critical mass through a multistage propagation process involving heterogeneous groups and knowledge bases. In line with the global trend, we observe the active presence of patient support and advocacy groups in the agenda-setting process. An effort to build the minimum critical mass is underway in which various groups are engaged in the propagation process. We find the emergence of diverse epistemic communities, too in this regard. As one would expect, the members of these communities comprise researchers, scientists of reputed universities and physicians from reputed tertiary level hospitals.

In addition, a considerable amount of knowledge seems to exist among the members of patient support and advocacy group and primary care physicians too. Unfortunately, however, the path dependence in the health policymaking structure in the country does not make it easy for these groups to participate in the policy deliberation process.^10^ The exclusion of site-specific, lived experiential knowledge from the deliberative process may have prompted some of the excluded groups to take the legal route, subsequently expediting the policy process through the judicial interventions. Though the Judiciary has been able to fast track the policy process, it has not yet helped achieve the desired level of participation, especially of the various informally organized groups and primary care physicians operating at the local levels. The Judiciary’s involvement to expedite the policy process may have also curtailed the scope of policy learning through trial and error. It may, eventually, prove to be counterproductive by eliminating a rich source of creating knowledge (through experience and trial-and-error) relevant for policymaking under uncertainty.

Skepticism regarding universal applicability and usefulness of ‘hard evidence’ such as RCT in policymaking is nothing new (see, for instance, Frieden, 2017). There is a solid rationale to move beyond such hard objective evidence; to adopt a community-based approach to solve problems, especially in rare diseases. In this field, where resource constraint and evidentiary vacuum are cited as challenges for effective policymaking, a community-driven, decentralized, problem-solving approach can be a game-changer. Such a framework can articulate how individual communities can nurture their success and develop plans of action to promote their scale-up in the absence of a centralized mechanism. All this raises important theoretical questions on the existing definition of epistemic community, perhaps, requiring broadening of its ambit to include the diverse (less formalized) forms of knowledge and experiences.

## Footnotes

1 Some authors argue that the distinction is sometimes overstretched, and the two approaches essentially share substantive common grounds among themselves. See Dhami et al. (2018).

2 Dhami et al. (2018) however claim that frequency interpretation cannot rule out all the errors in interpretation.

3 To deploy a robust RCT one must be able to have enough data, in a specific context, for effective randomization and probability interpretation. The data for a majority of rare diseases spreads thinly across contexts and places. These characteristics of the data reduce the scope for randomization, and ‘portability’ of results across contexts.

4 Unlike, for instance, the case of Covid-19, which attracted prompt policy attention across the globe due to the quick and severe public harm it causes.

5 The European Patient Advocacy Groups (ePAGs) is a case in point.

6 The National Institution for Transforming India (NITI) Aayog is currently the apex public policy think tank of the Government of India.

7 Note that the initiation of the policy was triggered by an order by the Delhi High Court.

8 At times, the basic science researchers too favour their demand.

9 For instance, organizing the social events such as ‘RACE for 7’, with the involvement of celebrities and prominent societal members. The first meeting, organized by a prominent patient advocacy group, even witnessed the presence of the charismatic former president of India Dr. A.P.J Kalam.

10 For instance, the past experiences of creating registries for diseases such as Cancer, Leprosy and is being utilized to frame the rare disease registry by the Indian Council of Medical Research. While such learning is essential, they do not adequately include the disease specific knowledge available with the patient advocacy groups, primary level clinicians, and basic science researchers-which are considered to be immensely important for policymaking in this domain.
